# Diet variation in a critically endangered marine predator revealed with stable isotope analysis

**DOI:** 10.1098/rsos.220470

**Published:** 2022-08-17

**Authors:** Courtney Ogilvy, Rochelle Constantine, Sarah J. Bury, Emma L. Carroll

**Affiliations:** ^1^ School of Biological Sciences, University of Auckland, Auckland 1010, New Zealand; ^2^ Institute of Marine Science, University of Auckland, Auckland 1010, New Zealand; ^3^ National Institute of Water and Atmospheric Research, Greta Point, Wellington 6021, New Zealand

**Keywords:** stable isotopes, diet, niche space, Bayesian mixing models, dolphin, *Cephalorhynchus*

## Abstract

Understanding the foraging ecology of animals gives insights into their trophic relationships and habitat use. We used stable isotope analysis to understand the foraging ecology of a critically endangered marine predator, the Māui dolphin. We analysed carbon and nitrogen isotope ratios of skin samples (*n* = 101) collected from 1993 to 2021 to investigate temporal changes in diet and niche space. Genetic monitoring associated each sample with a DNA profile which allowed us to assess individual and population level changes in diet. Potential prey and trophic level indicator samples were also collected (*n* = 166; 15 species) and incorporated in Bayesian mixing models to estimate importance of prey types to Māui dolphin diet. We found isotopic niche space had decreased over time, particularly since the 2008 implementation of a Marine Mammal Sanctuary. We observed a decreasing trend in ∂^13^C and ∂^15^N values, but this was not linear and several fluctuations in isotope values occurred over time. The largest variation in isotope values occurred during an El Niño event, suggesting that prey is influenced by climate-driven oceanographic variables. Mixing models indicated relative importance of prey remained constant since 2008. The isotopic variability observed here is not consistent with individual specialization, rather it occurs at the population level.

## 1. Introduction

The foraging ecology of animals can be influenced by variables such as prey distribution [1–3], predation risk [4–6], geographical location [7–9], local weather [10,11], sex [12–14] and reproductive status [15,16]. Understanding foraging ecology is important for gaining insight into trophic relationships and resource use of animals, with foraging success critical for survival. Knowledge of foraging activity and diet can inform conservation strategies and influence the outcomes of conservation initiatives. For example, supplemental feeding programmes can reduce seasonal mortality and increase population size [17,18], whereas identification and protection of foraging grounds could lead to increased reproductive success [19], and reduce anthropogenic-induced mortality [20–22]. Therefore, increasing our understanding of the foraging ecology of endangered species can be crucial to improving conservation outcomes.

Traditional methods used to gain insight into foraging ecology include direct observation of feeding events and stomach content analysis [23–26]. However, these methods are not well suited to many endangered species. Direct observation of feeding events relies on sightings of animals, which are often opportunistic and in remote locations, sometimes only accessible in certain seasons and times throughout the day leading to temporal and spatial biases. Stomach content analysis requires capturing or handling animals, or carcasses, and may give biased results due to differential prey residency times in the stomach [23]. A commonly used alternative approach to investigate foraging ecology that can overcome these limitations is stable isotope analysis [27]. Isotope ecology is based on the premise that the isotopic composition of a consumer's tissues will reflect that of its prey [23,28–30]. Specifically, stable nitrogen isotope ratios are indicators of trophic position [28,29,31,32], while carbon isotope ratios can indicate foraging location [30,33,34].

Stable isotope analysis is especially useful for assessing the diet of animals that live in habitats that make them difficult to observe and/or undergo large migrations. Analysis can be performed with a small amount of tissue and is minimally invasive [28]. The amount of time it takes for isotopes in the tissue of the consumer to be replaced with isotopes derived from diet is referred to as the ‘isotopic turnover rate’. This varies among species and tissue types; substrates such as skin and muscle are commonly used to integrate diet over broader time scales than is possible with stomach content analysis [23,28]. The most common application of stable isotope analysis in ecology is the characterization of trophic level [23,28,35]. Consumers are enriched in ^15^N relative to their prey, enabling nitrogen isotope ratios to determine trophic position [32,34,36]. During assimilation, the lighter isotope is preferentially excreted in a process known as trophic enrichment, and the consequent offset between consumer and prey is referred to as the trophic enrichment factor (TEF) [23,28,32,37,38]. Conversely, carbon isotopes are used to identify primary energy sources in a trophic network and can provide insight into foraging locations [34,36]. In marine species, foraging ecology is strongly influenced by the dynamic marine environment [39]. Carbon isotopes can differentiate between inshore and offshore habitats, or contribution of pelagic versus benthic prey sources to consumer diet [34,40–42]. Carbon isotopes also vary across a latitudinal gradient and are used to indicate foraging locations in animals that undertake long migrations [28,41,43,44].

Coastal marine environments are some of the most ecologically and socioeconomically important on the planet, but their proximity to land utilization exposes them to anthropogenic impacts. Near-shore zones are at risk of degradation through runoff [45], and apex marine predators are at risk of fishing-related mortality, for example, through entanglement in fishing-gear [46,47]. Poorly managed commercial fishing can also affect abundance and distribution of targeted species [48] and their predators such as cetaceans [49,50]. The rate and distribution of primary production in coastal environments are also altered by anthropogenic impacts, particularly climate change. The effect this has on marine food web structure has been linked to changes in foraging ecology of apex predators (e.g. cetaceans, pinnipeds and piscivorous sharks) [45,51–53].

**Figure 1.**
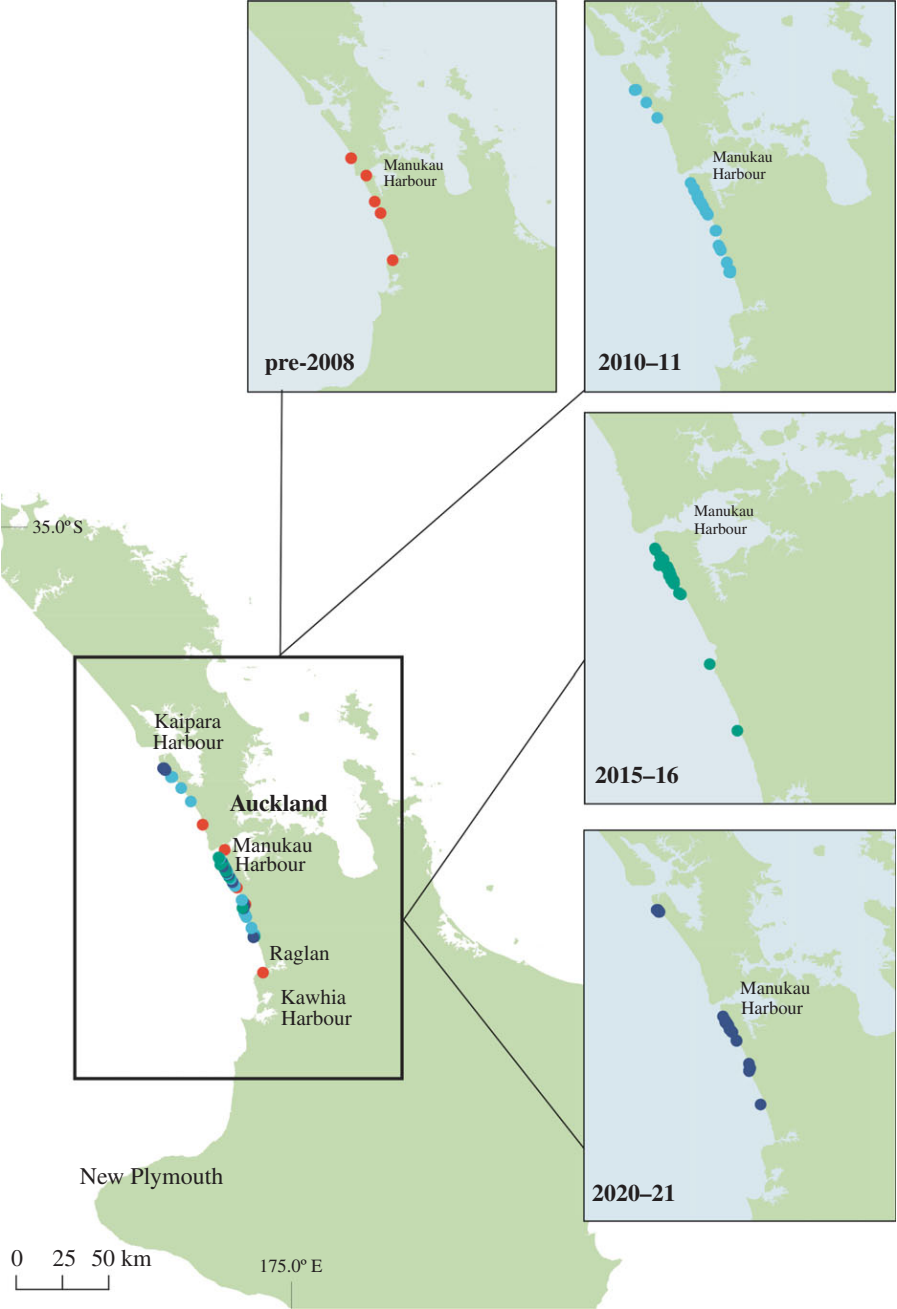
Map showing the distribution of Māui dolphins on the west coast of the North Island, New Zealand, and the location of Māui dolphin biopsy samples used in stable isotope analysis, coloured by year of sample collection.

The most endangered marine dolphin in the world lives in the near-shore waters of Auckland (Tāmaki Makaurau), the largest city in Aotearoa New Zealand (hereafter New Zealand). The critically endangered Māui dolphin (*Cephalorhynchus hectori maui*) [54] is a sub-species of Hector's dolphin (*Cephalorhynchus hectori hectori*) and is endemic to New Zealand, with an estimated abundance of 54 individuals aged greater than one year (95% CI = 48, 66) [55]. They are primarily found within a 40 km stretch of Auckland's west coast (figure 1) [55–58]. Gillnet fisheries introduced in the 1960s caused a population decline up until the mid-2000s through by-catch mortality [58,59]. Since 2008, a Marine Mammal Sanctuary (MMS) encompassing the core distribution of Māui dolphins has been in place to prevent further population decline. Within the MMS, recreational and commercial set-netting and trawling between 4 and 12 nautical miles from shore are prohibited [60]. Restricted fisheries activity in other locations has resulted in ecosystem restoration and increases in abundance of previously depleted species [61–64].

Information on Māui dolphin diet is limited to one publication describing the stomach contents of just two beachcast individuals [65]. These dolphins had consumed red cod (*Pseudophycis bachus*), ahuru (*Auchenoceros punctatus*), sole (*Peltorhamphus* spp.) and flounder (*Rhombosolea plebia*). Hector's and Māui dolphins preferentially target fish less than 10 cm in length, including juveniles and prefer to inhabit turbid waters [56,65,66]. The low population abundance of Māui dolphins and preference for turbid waters mean stomach content analyses, direct observations and scat collection were not practical tools for inferring diet of this sub-species.

Given these limitations and the lack of existing data, the goal of this project was to better understand the foraging ecology of Māui dolphins and assess how their diet has changed over time. We addressed knowledge gaps in their foraging ecology by analysing ∂^13^C and ∂^15^N values in skin samples. The majority of these samples came from long-term genetic monitoring work [41], meaning that each sample was associated with a unique DNA profile. This allowed us to understand how the diet of Māui dolphins had changed over time on both a population and individual level.

## 2. Material and methods

### 2.1. Sample collection

Māui dolphin samples analysed in this study (table 1) are from skin biopsy samples collected from live individuals (*n* = 96) and skin samples collected from dead beach cast dolphins (*n* = 5) with permission from the University of Auckland Animal Ethics Committee and New Zealand Department of Conservation, in consultation with mana whenua (Māori, the Indigenous people of the region). As previously described [57,59], skin biopsy samples were collected during boat surveys using a small, lightweight biopsy dart fired from a modified veterinary capture rifle (PaxArms NZ Ltd) [67]. Biopsy samples were collected between 2001 and 2021, primarily during the austral summer–autumn (January–March). Calves less than 1 year old (less than half the length of an adult and in close association with their mother [68]) were excluded from biopsy sampling. Samples were stored in 70–90% ethanol at −20°C until required for stable isotope analysis.

**Table 1.** Māui dolphin skin samples collected for stable isotope analysis, representing unique individuals per year, grouped by year of sample collection and genetically determined sex.

year	male	female	total sample size (*n*)
1993	0	1	1
2001	3	0	3
2002	1	0	1
2003	4	0	4
2006	0	1	1
2007	0	1	1
2010	8	6	14
2011	7	9	16
2015	6	8	14
2016	5	11	16
2020	4	10	14
2021	8	8	16
total	46	55	101

As the primary reason for collecting samples was to undertake genetic monitoring of the Māui dolphin, each sample had an associated DNA profile. This profile was comprised of genetically identified sex, mitochondrial DNA haplotype and microsatellite genotype (up to 21 microsatellite loci [55]). This allowed us to track the change in isotope values of individuals across time.

Fish specimens identified as potential prey or indicators of trophic level (hereafter referred to as potential prey) [56,65] were obtained for stable isotope analysis. Fish were caught within the current known primary distribution of Māui dolphins along the west coast of the North Island (Te Ika-a-Māui), and off the coast of New Plymouth (Ngāmotu), between 2012 and 2021 (electronic supplementary material, table S1). A small section of dorsal muscle tissue was removed from each fish between the head and the dorsal fin (approximately 5 cm × 5 cm × 1 cm depth) and frozen at −80°C until required for stable isotope analysis.

### 2.2. Stable isotope analysis

Lipids are depleted in ^13^C relative to other types of tissue such as protein and carbohydrates. Therefore, any material rich in lipids has a lower ∂^13^C value relative to other tissue types [33,69]. The difference in lipid content of individual organisms has the potential to confound the interpretation of ∂^13^C isotope ratios. To mitigate the effect of ^13^C depleted lipids, all dolphin and fish samples were lipid extracted prior to stable isotope analysis following previously published methodology [70]. The majority of the stable isotope analyses were carried out at Isotrace NZ Ltd, with a small number of samples analysed at the National Institute of Water and Atmospheric Research (NIWA) Ecological and Environmental Stable Isotope Analytical Facility. At Isotrace NZ Ltd, nitrogen and carbon isotopes were assayed by combustion of the whole material, to N_2_ and carbon dioxide (CO_2_) gas in a Carlo Erba NC2500 elemental analyser (CE Instruments, Milan) linked to a Europa Scientific ‘20/20 Hydra’ (Europa Scientific, UK) continuous flow isotope ratio mass spectrometer (CF-IRMS). At the NIWA laboratory, stable isotope analyses were carried out on a Delta V Plus CF-IRMS linked to a Flash 2000 elemental analyser using a MAS 200 R autosampler (Thermo-Fisher Scientific, Bremen, Germany). Stable isotope ratios are reported in ∂ notation:

$$\partial^{X}=\left[(R_{\mathrm{sample}}/R_{\mathrm{standard}})-1\right]\times 1000$$

where *X* is the isotope of interest (^13^C or ^15^N), and *R* is the ratio of heavy to light isotope (e.g. ^13^C/^12^C). IRMS software calculated ∂^15^N values against the international standard atmospheric N_2_, carbon isotope values were calculated against Carrara Marble NSB-19 (National Institute of Standards and Technology, Gaithersberg, MD, USA), which, in turn, was calibrated against the original Pee Dee Belemnite limestone standard and corrected for ^17^O. Details of standards and normalization processes are provided in the electronic supplementary material, S1.

At Isotrace, analytical precision for ∂^13^C and ∂^15^N was assessed via analyses of in-house reference materials for each run, which were stringently calibrated against international standards (e.g. USGS-40, USGS-41 and EDTA-OAS), and was measured as 0.1‰ for ∂^13^C and 0.2‰ for ∂^15^N (SD). At NIWA, international standards used were USGS-40, IAEA-N2 and IAEA-CH-6, and precision was measured as 0.1‰ for ∂^15^N and 0.2‰ for ∂^13^C.

### 2.3. Controlling for atmospheric changes to ∂^13^C values

We investigated temporal trends in isotopic values of Māui dolphins. However, ∂^13^C values in the biosphere have decreased exponentially since the beginning of the industrial revolution due to the burning of fossil fuels. The CO_2_ introduced into the biosphere from fossil fuel burning has a lower ∂^13^C value than background atmospheric CO_2_. This difference is termed the ‘Suess effect’ [71]. Due to the increased concentration of aqueous CO_2_ in the ocean since the beginning of the industrial revolution, the Suess effect also influences the ∂^13^C values of the world's oceans [72]. A correction for the oceanic Suess effect (0.011‰ yr^−1^) [73] was applied to the stable isotope values of Māui dolphin and potential prey samples, using the average year between the years of sampling of potential prey (2017) as a reference [74], to allow the comparison of ∂^13^C values from specimens from different time periods.

### 2.4. Tests for heterogeneity of isotope values by time and demographic state

Carbon and nitrogen isotope values for Māui dolphin and prey samples were plotted in R v. 4.0.0 to visually inspect the data for any trends [75]. Statistical analyses were carried out in R v. 4.0.0. The Shapiro–Wilk test of normality was used to assess the distribution of ∂^13^C and ∂^15^N values. The Kruskal–Wallis and *post hoc* Dunn's multiple comparison tests were used to assess differences in ∂^13^C and ∂^15^N values of dolphin skin with respect to year of sampling and sex. Hierarchical cluster analysis (Ward's minimum variance method) was used to segregate dolphin data into two distinct groups, and to combine potential prey data into five distinct groups.

### 2.5. Isotopic niche space analyses

We assessed differences in the isotopic niche space of Māui dolphins over time, including before and after the implementation of the MMS (2008), using the Stable Isotope Bayesian Ellipses (SIBER, v. 2.1.6) package in R [76]. Bivariate ellipses of ∂^13^C and ∂^15^N values with 95% confidence intervals were used to estimate the isotopic niche space for dolphins sampled in 1993–2008, 2010–2011, 2015–2016, 2020 and 2021. Bayesian standard ellipse areas (SEA_B_) were plotted using SIBER to show niche overlap and changes in isotopic niche space between time periods. Niche area is reported as ‰^2^ and was estimated by running two Markov chain Monte Carlo (MCMC) chains each comprising 1 000 000 iterations, following 100 000 burn-ins, implemented in rjags [77]. A Gelman–Rubin diagnostic test was used to assess convergence, indicated by a scale reduction factor less than 1.1 [78].

### 2.6. Bayesian mixing model analyses

A common application of stable isotope analysis in foraging ecology is the use of Bayesian mixing models to estimate the proportional contributions of prey sources to the isotopic signature of the consumer, which reflects their assimilated diet. Bayesian mixing models use isotope values and TEFs to estimate the assimilated diet of the consumer, while accounting for uncertainty in isotopic variability of consumers and multiple sources [79–81]. To estimate the proportional contribution of potential prey (sources) to overall diet of Māui dolphins (consumers), Bayesian stable isotope mixing models were implemented using the MixSIAR package in R [82]. Mixing models containing more than seven sources are unlikely to produce accurate and meaningful diet estimations [79]. Mixing models are also unable to differentiate between sources with similar isotopic values [80]. The different species of potential prey specimens collected had similar isotope ratios, indicating they would probably occupy a similar isotopic niche space and would not be sufficiently distinct for mixing models to resolve. Consequently, the mean isotope values for each species (*n* = 166; 15 species) were grouped using a hierarchical cluster analysis (*k* = 5; Ward's minimum variance method) into five isotopically distinct sources (electronic supplementary material, figures S1 and S2). The mean and associated error of each source was determined by averaging the mean ∂^13^C and ∂^15^N values for each species forming the cluster (see electronic supplementary material, table S1, for source membership of each species).

To distinguish sources based on their ∂^15^N values, trophic levels were calculated for each source. The trophic level calculation used was

$$\mathrm{TL}=2+\frac{\left(\partial^{15}N_{\mathrm{specimen}}-\;\partial^{15}N_{\mathrm{primary}\;\mathrm{consumer}}\right)}{1.68}\;$$

where ∂^15^N_specimen_ represents the nitrogen isotope ratio of the potential prey species, ∂^15^N_primary consumer_ represents the nitrogen isotope ratio of the baseline, green-lipped mussels (*Perna canaliculus*), 2 is the assumed trophic position of the baseline consumer and 1.68 is the estimated ∂^15^N enrichment per trophic level for Māui dolphins [83,84]. To determine the trophic level of each source, we averaged the trophic level of each species comprising that source. For ease of reading, the resultant trophic levels were categorized into three tiers.

Sources were also described based on their ∂^13^C values and approximate distance from shore. In order of ‘nearest to shore’ and ‘highest ∂^13^C value’, sources were defined as ‘inshore’, ‘shelf’ (i.e. continental shelf-associated) and ‘pelagic’. Finally, the habitat type of each species comprising the mean isotopic value for each source was included (demersal, benthopelagic and pelagic). The Kruskal–Wallis and *post hoc* Dunn's tests were used to confirm the five sources were significantly different and isotopically distinct.

**Figure 2.**
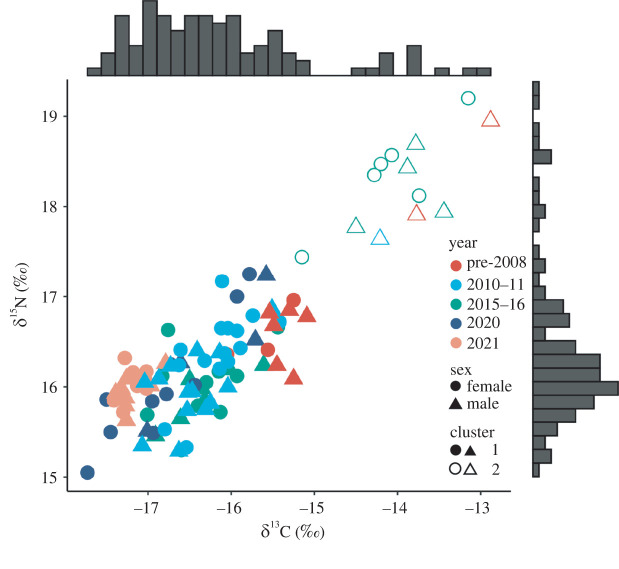
Distribution of ∂^13^C and ∂^15^N values from 101 Māui dolphin skin samples collected between 1993 and 2021. Values are grouped by year of sample collection (colour), sex (shape) and cluster (fill). Frequency distributions of ∂^13^C and ∂^15^N are shown as marginal histograms.

We visually examined isotope mixing polygons (mixing space) to ensure that consumer data were within the mixing space [80,85]. Due to the broad distribution of consumer data, a substantial proportion of consumer data points were outside of the mixing space. A hierarchical cluster analysis (*k* = 2, Ward's minimum variance method) was used to group the consumer data into two sets (Cluster 1 and Cluster 2; figure 2), and as a result, we assessed two sets of mixing models with different combinations of consumer and source data.

‘Set A’ contained consumers from Cluster 1 and five sources. We ran five mixing models in Set A: one for each period of interest (pre-2008, 2010–2011, 2015–2016, 2020 and 2021). ‘Set B’ contained consumers from Cluster 2 and six sources. For Set B, we undertook a sensitivity analysis to understand the potential importance of putatively absent source data. The mixing polygon was artificially extended to include the higher ∂^13^C and ∂^15^N values of Cluster 2 consumers, by the addition of a proxy source [86,87]. The value and associated error of the proxy source were determined using empirical data by calculating the average difference in ∂^13^C and ∂^15^N values between sources and applying this to the most positive ∂^13^C and ∂^15^N values observed in the source data. To sufficiently extend the mixing space to include consumer data from Cluster 2, the average difference in ∂^13^C and ∂^15^N values was applied to the most positive ∂^13^C and ∂^15^N values three times. To test the potential importance of the proxy source for these consumers, we combined all year groups in Cluster 2 and ran one model. The TEFs used in both Set A and Set B for ∂^13^C and ∂^15^N were 1.60 ± 0.09 and 1.68 ± 0.11, respectively [83]. We chose these TEFs due to the similarity in lipid content of our potential prey samples and those used to calculate the TEF in [83]. The C : N ratio was used as a proxy for lipid content to select the TEF from the diet with the most similar C : N ratio and overall ∂^13^C and ∂^15^N values to our samples.

Each mixing model was run with three MCMC chains, each comprising 100 000 iterations where the first 50 000 iterations were discarded. Sampling was conducted at intervals of 50 iterations. Source data had non-informative prior distributions. A process × residual error model structure was used [88]. The Gelman–Rubin diagnostic test was used to assess model convergence, indicated by a scale reduction factor less than 1.1 [78].

All R code used in isotopic niche space and Bayesian mixing model analyses is available on GitHub: https://github.com/courtneyogilvy/stable-isotope-analysis [89] and has been archived within the Zenodo repository: https://doi.org/10.5281/zenodo.6835828.

## 3. Results

### 3.1. Stable isotope ratios of Māui dolphin skin samples

Māui dolphin samples (*n* = 101), collected from 1993 to 2021, had an overall mean ∂^13^C value and standard error (s.e.) of −16.1 ± 0.1‰ (range: −17.7 to −12.9‰) and a mean ∂^15^N of 16.4 ± 0.1‰ (range: 15.1 to 19.2‰; table 2). The ∂^13^C and ∂^15^N distributions of the combined dataset were not normally distributed (figure 2; Shapiro–Wilk W-test: ∂^13^C: *p* < 0.0001; ∂^15^N: *p* < 0.00001).

**Table 2.** Mean, median, minimum and maximum values of ∂^13^C and ∂^15^N values in Māui dolphin skin samples collected from 1993 to 2021, organized by year of sample collection. s.e. is standard error.

year group	*n*	∂^13^C				∂^15^N			
		mean ± s.e. (‰)	median (‰)	min. (‰)	max. (‰)	mean ± s.e. (‰)	median (‰)	min. (‰)	max. (‰)
pre-2008	11	−15.1 ± 0.3	−15.3	−16.1	−12.9	16.9 ± 0.3	16.8	16.1	19.0
2010–2011	30	−16.2 ± 0.1	−16.3	−17.1	−14.2	16.2 ± 0.1	16.3	15.3	17.6
2015–2016	30	−15.6 ± 0.2	−16.1	−17.0	−13.2	16.8 ± 0.2	16.1	15.3	19.2
2020	14	−16.7 ± 0.2	−16.8	−17.7	−15.6	16.1 ± 0.2	16.0	15.1	17.3
2021	16	−17.2 ± 0.0	−17.3	−17.4	−16.8	16.0 ± 0.1	16.0	15.6	16.3
males	46	−16.0 ± 0.2	−16.3	−17.4	−12.9	16.5 ± 0.1	16.1	15.3	19.0
females	55	−16.2 ± 0.1	−16.3	−17.7	−13.2	16.4 ± 0.1	16.2	15.1	19.2
total	101	−16.1 ± 0.1	−16.4	−17.7	−12.9	16.4 ± 0.1	16.2	15.1	19.2

### 3.2. Tests for heterogeneity of isotope values by time and demographic state

There were no significant differences in ∂^13^C and ∂^15^N values between individual years from 1993–2008, 2010 and 2011 and 2015 and 2016 (Kruskal–Wallis test; electronic supplementary material, table S2) so these years were pooled together as ‘pre-2008’, ‘2010–2011’ and ‘2015–2016’, respectively (figure 2), and treated collectively in statistical analyses. There were no significant differences in ∂^15^N values for the years 2020 and 2021 but ∂^13^C values were significantly different so these were treated separately. All sampling year groups were statistically significantly different with respect to ∂^13^C and ∂^15^N (∂^13^C: K–W *χ*^2^ = 52.8, *p* ≤ 0.00005; ∂^15^N: K–W *χ*^2^ = 13.6, *p* < 0.05). *Post hoc* Dunn's multiple comparisons indicated that ∂^13^C and ∂^15^N values for samples collected before 2008 were significantly higher than ∂^13^C and ∂^15^N values for samples collected in 2010–2011, 2015–2016, 2020 and 2021. ∂^13^C values for the years 2015–2016 were significantly higher than ∂^13^C values for samples collected in 2010–2011, 2020 and 2021 (electronic supplementary material, tables S3 and S4). There were no significant differences in ∂^13^C and ∂^15^N between males and females (∂^13^C: K–W *χ*^2^ = 0.60, *p* = 0.44; ∂^15^N: K–W *χ*^2^ = 0.09, *p* = 0.76).

### 3.3. Individual-level changes over time

**Figure 3.**
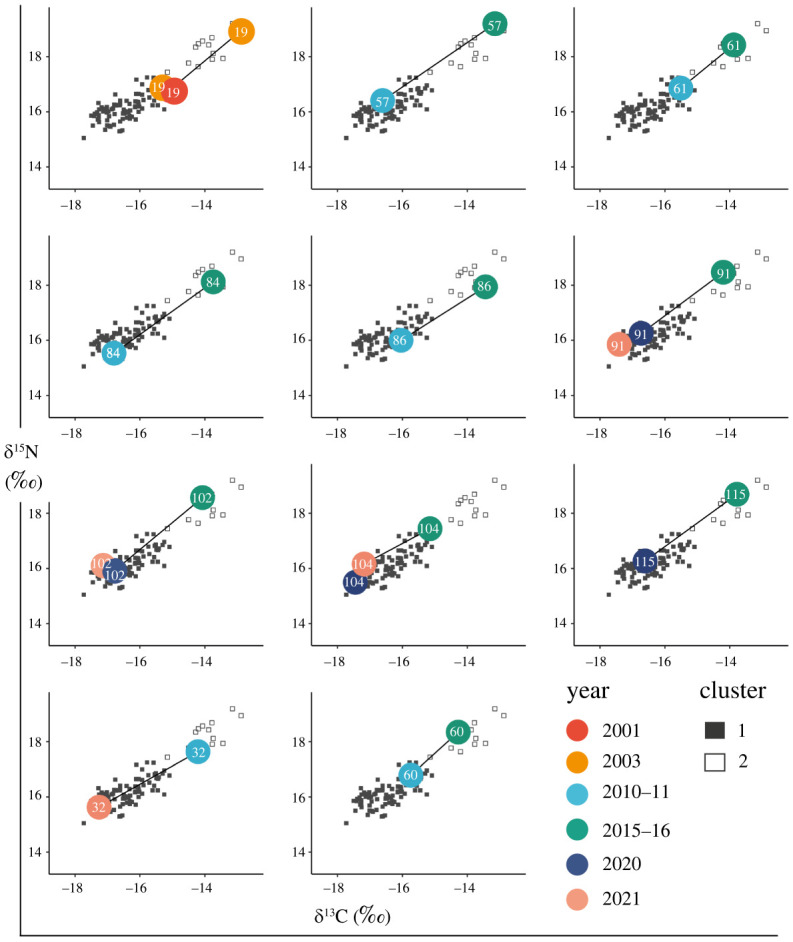
Combined resampling data of Cluster 2 individuals. Each plot represents an individual from Cluster 2 which was sampled in more than 1 year; ∂^13^C and ∂^15^N values are coloured by year with resampling indicated by a black line. Individual ID number is shown for that datapoint in each coloured circle. Consumers (Māui dolphins) in Clusters 1 and 2 are represented by closed and open grey squares, respectively.

Nearly all isotope values in Cluster 2 (*n* = 11) were from individuals who had been biopsied in at least one other year based on DNA profile data. For the Cluster 2 individuals who were biopsied again in either 2010–2011, 2020 or 2021, ∂^13^C and ∂^15^N values for these years were within the range of Cluster 1 (figure 3).

### 3.4. Māui dolphin isotopic niche space

**Figure 4.**
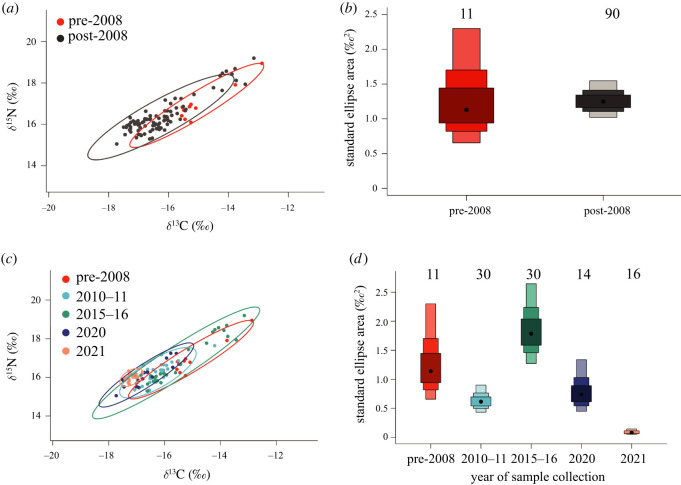
(*a*) Isotopic niche space indicated by 95% CI bivariate ellipses of Māui dolphin skin samples collected before 2008 (red, *n* = 11) and after 2008 (grey, *n* = 90). (*b*) Bayesian standard ellipse area (SEA_B_) of isotopic values in Māui dolphin skin samples collected before 2008 (*n* = 11) and after 2008 (*n* = 90). The black dot in the centre represents the mode, and the shaded boxes represent the 50%, 75% and 95% credible intervals from dark to light grey. Sample size is displayed above each box. (*c*) Isotopic niche space indicated by 95% CI bivariate ellipses of Māui dolphin skin samples*,* grouped by the year of sample collection. (*d*) Bayesian standard ellipse area (SEA_B_) of isotopic values in Māui dolphin skin samples grouped by year of sample collection. The black dot in the centre represents the mode, and the shaded boxes represent the 50%, 75% and 95% credible intervals from dark to light. Sample size is displayed above each box.

The SIBER analysis of Māui dolphin skin samples collected before (*n* = 11) and after (*n* = 90) 2008, when the MMS was established, indicated a contraction in isotopic niche space of the Māui dolphins sampled after 2008 (figure 4*a,b*).

The SIBER analysis showed isotopic niche space was similar in the years 2010–2011 and 2020 (figure 4*c*,*d*). We observed a decreasing trend in niche space after 2008, except for 2015–2016 where niche space increases to a similar size observed in pre-2008 (figure 4*d*).

### 3.5. Dietary estimations—model Set A

**Figure 5.**
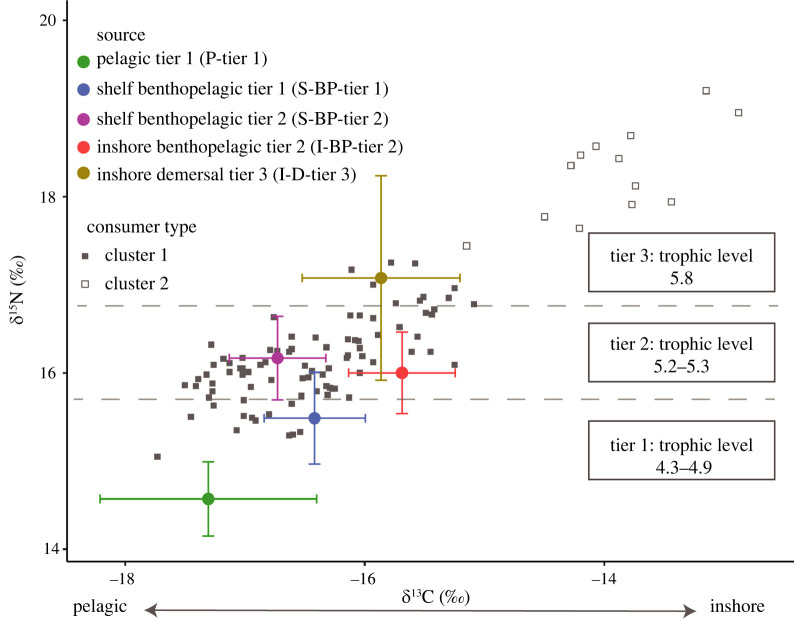
MixSIAR isotope mixing polygon for Set A mixing models, showing source (prey) and consumer (Māui dolphin) ∂^13^C and ∂^15^N values. Isotope values of Māui dolphins are grouped according to cluster (Cluster 1: closed grey squares; Cluster 2: open grey squares). Sources are grouped by colour and have been corrected for trophic enrichment (∂^13^C: 1.60 ± 0.09; ∂^15^N: 1.68 ± 0.11) [83]. s.d. for each source are represented by error bars.

Bayesian mixing models assessed if the proportional contributions of prey items to Māui dolphin diet had changed over time. The trophic levels calculated for each source ranged from 4.3 to 5.8 (electronic supplementary material, table S1). Due to the similarity in trophic level of the sources, we categorized each source into three trophic tiers (figure 5). Sources were significantly different from each other (Kruskal–Wallis test; *p* < 0.05).

**Figure 6.**
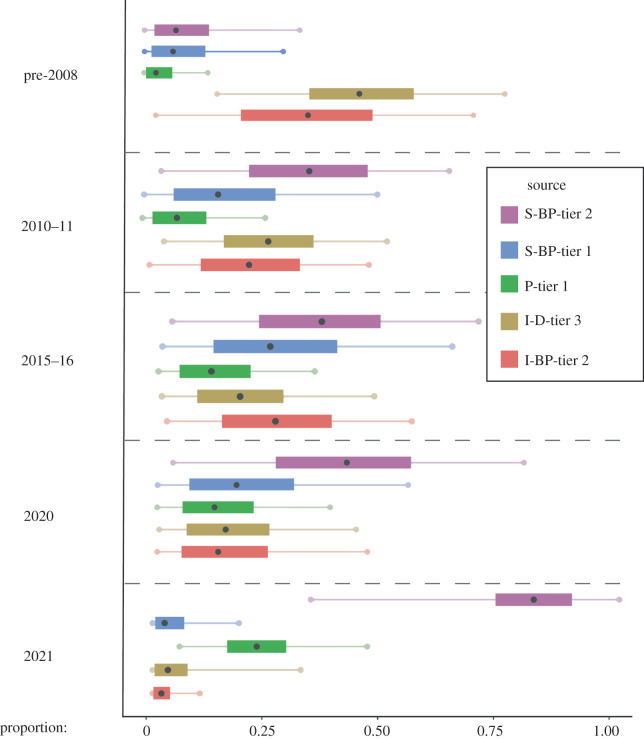
MixSIAR model outputs showing estimates of diet composition for Māui dolphins in Cluster 1; sampled before 2008, in 2010–2011, 2015–2016, 2020 and 2021. The median is represented by the black dot, and coloured boxes and lines represent 50% and 95% credibility intervals, respectively. Each segment within dotted lines represents the model output for the period of interest. Source contributions are represented by colour. Source labels are I-BP-tier 2: inshore benthopelagic tier 2; I-D-tier 3: inshore demersal tier 3; P-tier 1: pelagic tier 1; S-BP-tier 1: shelf benthopelagic tier 1; S-BP-tier 2: shelf benthopelagic tier 2.

A mixing model was run for each statistically different year group (pre-2008, 2010–2011, 2015–2016, 2020 and 2021) to assess how dietary contributions had changed over time. When corrected for trophic enrichment, the isotope ratios of potential prey overlapped with Māui dolphin isotope ratios belonging to Cluster 1 (figure 5). Isotope ratios of consumer and source data plotted together in an ‘isospace’ plot showed Māui dolphin samples from Cluster 1 lay within the mixing polygon, defined by the mean and associated error of each of the five sources. We found that for the years 2010–2011, 2015–2016, 2020 and 2021, the greatest contributor to Māui dolphin diet was continental shelf-associated, benthopelagic prey with trophic level 5.2 to 5.3. Importantly, this indicates that for consumers in Cluster 1, diet composition has not changed substantially since 2008. By contrast, for the years prior to 2008, the greatest contributor to diet was inshore, demersal prey of trophic level 5.8 (figure 6).

### 3.6. Dietary estimations—model Set B

**Figure 7.**
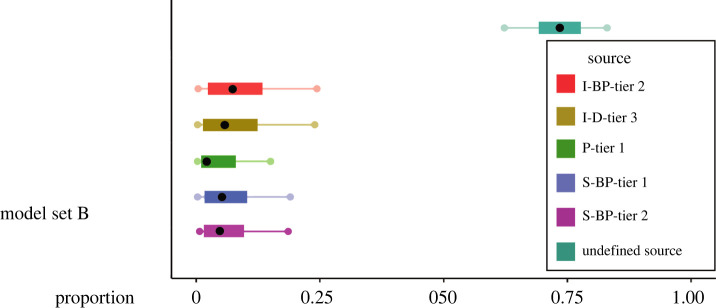
MixSIAR model output for model Set B, showing estimates of diet composition for Māui dolphins in Cluster 2. The median is represented by the black dot, and coloured boxes and lines represent 50% and 95% credibility intervals, respectively. Abbreviated source labels are I-BP-tier 2: inshore benthopelagic tier 2; I-D-tier 3: inshore demersal tier 3; P-tier 1: pelagic tier 1; S-BP-tier: shelf benthopelagic tier 1; S-BP-tier 2: shelf benthopelagic tier 2.

Māui dolphin samples belonging to Cluster 2 were enriched in ^13^C and ^15^N and consequently required an extension of the mixing polygon to run a mixing model analysis. The values used for the hypothetical ‘undefined source’ were −14.86 and 17.65 for ∂^13^C and ∂^15^N, respectively. This source had the greatest contribution to overall diet composition of consumers in Cluster 2 with a median contribution of 69.3% (figure 7).

## 4. Discussion

This study documents ∂^13^C and ∂^15^N values in the skin of a critically endangered coastal predator, the Māui dolphin, collected over a period of 28 years (1993–2021). Here we reveal that the diet of this population has changed substantially over the 28-year study period, particularly since the implementation of the MMS. The associated DNA profiles of each sample indicate the changes observed here are not occurring at the individual level but instead reflect population-level variation.

### 4.1. Temporal differences in foraging ecology

Temporal changes in foraging ecology can be due to ontogeny and development [90,91], individual specialization [92,93], ecosystem baseline changes [94,95] or changes in prey availability and preferences [96,97]. Here, we observed temporal changes in foraging ecology reflected by fluctuations in isotope values of Māui dolphins. Nitrogen isotope values in Māui dolphin samples showed a slight decreasing trend with year, indicating a shift in diet or changes in the ecosystem that have resulted in a shift of the isotopic baseline of primary production. The decreasing trend observed here was unexpected as other coastal dolphin species have shown increasing or stable ∂^15^N values with time [98,99]. Increases in ∂^15^N over time are likely due to the increased urbanization of coastal areas with high levels of terrestrial nitrogen runoff from fertilizer [100,101]. Given the near-shore habitat of Māui dolphins and the proximity to Auckland, it was surprising to see a decrease in ∂^15^N values with time. Some species exhibit patterns in ∂^15^N values associated with age [102–104], but this is an unlikely explanation for the decreasing ∂^15^N trend observed here. In other marine species such as Antarctic fur seals (*Arctocephalus gazelle*) [104], beluga whales (*Delphinapterus leucas*) [102] and Weddell seals (*Leptonychotes weddellii*) [103], increases in ∂^15^N values with age are associated with the consumption of higher trophic level prey compared to younger animals. Weddell seals also have increasing ∂^13^C values with age, suggesting a shift in foraging location of older animals to more coastal habitats or the consumption of more benthic/demersal prey compared to younger animals. It is possible the decreasing ∂^15^N trend we see is due to a shift in prey resource, as has been observed in other species [105–107]. Nitrogen isotope values in beluga whales decreased over time and are thought to represent a shift to a new prey resource, where the new prey has a relatively lower ∂^15^N value compared to earlier prey [102]. Some seabirds have also undergone a shift in prey to consume greater proportions of fish which have lower ∂^15^N values [108].

The productivity of marine ecosystems can be inferred by ∂^13^C values; more positive values indicate near-shore, productive regions whereas more negative (depleted) values are indicative of less productive, offshore regions [28]. A decline in ∂^13^C values over time can be attributed to a reduction in the net primary productivity of an ecosystem, or a shift in foraging activity to more pelagic prey which are typically depleted in ^13^C [34,109], or a combination of both. Decreasing ∂^13^C values can also be attributed to the oceanic Suess effect [72,110], but as we have corrected our data to account for the Suess effect, we have removed that possibility. Here we see ∂^13^C values decreased by 0.08‰ yr^−1^ over the course of the study. In other species [102,110–112], decreases in ∂^13^C values over time have been attributed to dietary niche shifts and/or shifts in isotopic baseline of the ecosystem over time. For example, this has been observed in top marine predators such as polar bears (*Ursus maritimus*), where significant decreases in ∂^13^C over time corresponded with a shift from benthic to pelagic prey sources associated with changes in temperature and sea ice cover [111].

### 4.2. Influence of climate and oceanographic conditions on foraging ecology

While we see a general pattern of decreasing ∂^15^N and ∂^13^C values over time (figure 2), this is nonlinear and several fluctuations were observed throughout the study period, particularly in the years 2015–2016 (figure 4*d*). Temporal fluctuations in the diet of generalist predators are not unusual and have been reported in other marine predators [43,102,111–114]. Humpback whales (*Megaptera novaeangliae*) in the California Current System exhibited variation in isotope values which were reflective of major shifts in oceanographic and ecological conditions associated with upwelling events and shifts in prey availability [112]. Likewise, baleen whales in the North Atlantic have exhibited distributional shifts associated with climate-driven changes in oceanographic conditions [115]. An example of climate-driven changes to oceanographic conditions is the El Niño Southern Oscillation (ENSO). ENSO conditions are likely to influence prey distribution and cause dolphins to forage outside of typical areas to find adequate prey sources [116]. Indo-Pacific bottlenose dolphins (*Tursiops aduncus*) resident in west Australia exhibit variations in abundance and distribution associated with ENSO events. Throughout a 6-year study, abundance was recorded to be lowest during the year which coincided with the only El Niño event during the study period [116]. A similar phenomenon was observed at Santa Catalina Island, USA where the abundance of pilot whales (*Globicephala macrorhynchus*) decreased by several hundred individuals during the 1982–1983 ENSO [117].

Given fluctuation in isotope values over time is not uncommon in cetaceans, we were not surprised to observe it here. However, the greatest change in isotope values is observed in 2015–2016 (figure 4*d*) suggesting a substantial shift in prey availability around this time. In 2015–2016, the largest El Niño event in 145 years impacted the Pacific region [118]. El Niño conditions in New Zealand lasted from June 2015 to April 2016 [119]. Given that Māui dolphin samples were collected between January and March and the isotopic turnover of skin in similar cetaceans is estimated to be between two and four months [83], the isotope values here represent prey consumed during the El Niño event. ENSO may alter availability of prey through changing ocean productivity [120], but there is limited information to describe how prey from the west coast of the North Island is affected by ENSO conditions. However, ENSO has been suggested as a cause of altered prey availability and foraging in New Zealand sea lions (*Phocarctos hookeri*) [121] and many species of penguin [122]. Consequently, it is possible Māui dolphin prey also shifted during this period, and the dolphins had to temporarily emigrate, similar to the bottlenose dolphins of west Australia [116]. Furthermore, Māui dolphins target juvenile fish [65,123]. Juvenile red cod which are consumed by Māui dolphins [65] are vulnerable to environmental factors influencing recruitment and have increased recruitment in colder years [124]. This is further evidence to support the theory that preferred Māui dolphin prey are affected by ENSO events.

Through the assessment of recapture histories for individuals sampled in more than one year, we determined that those with higher ∂^15^N and ∂^13^C values in 2015–2016 had lower values in other years (2010–2011, 2020 and/or 2021; figure 3). This suggests the variation observed is not due to individual specialization and is occurring at the population level. A more likely cause of the variation in 2015–2016 is a substantial shift in prey due to a change in oceanographic conditions associated with ENSO events and/or ingestion of the same prey type from a region of elevated ∂^15^N and ∂^13^C baseline.

### 4.3. Individual specialization and sex-related differences in foraging ecology

Foraging ecology is often assessed at the species level, and previously, it was thought that individuals were ecologically equivalent [92]. However, many mammals and birds exhibit individual specializations in foraging strategy [92,125,126]. Individual specialization can be a result of age or sex-related differences [12,90,127,128], but intraspecific variation can still be observed even after these have been accounted for. Individual specialization has been observed in top marine predators such as seabirds [92], sharks, [129], sea lions [130] and dolphins [131]. Female elephant seals (*Mirounga leonina*) of the West Antarctica Peninsula exhibit a high degree of individual specialization, reflected by low intra-individual variability in ∂^13^C values with respect to population variability [132]. The isotope values of Māui dolphins are inconsistent with individual specialization occurring here. For individuals sampled more than once across the study period, the variation moves with at least a portion of the population rather than with the individual. The individuals that displayed high ∂^13^C and ∂^15^N values in 2015–2016 and were then sampled again in other years all followed a similar pattern, with isotope values in other years falling back within the normal range of the population.

Sex-related differences in foraging strategy are often associated with sexual dimorphism [133]. This is particularly evident in larger seabirds [134] and pinnipeds [135,136]. We found no significant differences in the isotope values of males and females, indicating they consume similar prey and no sex-dependent foraging strategies are occurring. Sex segregation among social groups varies within the *Cephalorhynchu*s genus. The Hector's dolphin of Banks Peninsula (Te Pātaka-o-Rākaihautū) exhibits a high degree of sex segregation in groups which is reflected in the lower ∂^15^N values of males compared to females [123,137] whereas the Commerson's dolphin (*Cephalorhynchus commersonii commersonii*) off the coast of Tierra del Fuego, Argentina do not display any differences in isotopic values between sexes [138]. There is no evidence to suggest that male and female Māui dolphins are segregated by sex, although this may have happened when they were more abundant and the current social aggregations are the consequence of a social Allee effect [58,139].

We assessed the recapture histories of dolphins sampled in 2015–2016, to determine if this effect was age-related. While the exact age of individual dolphins was unknown, it was possible to gain an estimate of minimum age from recapture histories and/or to determine if each individual was of a similar age. We identified nine dolphins in the 2015–2016 group that had been sampled more than once, either in 2010–2011 or 2020–2021. This suggested the difference in age of individuals in 2015–2016 is up to 10 years, and consequently an age-related effect is an unlikely cause of the variation observed in these isotope values. An alternative hypothesis to explain the higher ∂^13^C and ∂^15^N values of these dolphins is nutritional stress. Animals experiencing nutritional stress can have higher ∂^15^N values due to reduced nutrient intake [140–143]. However, the degree to which ∂^13^C and ∂^15^N isotope values are effective indicators is unclear, with some studies reporting a reduction in ∂^13^C and ∂^15^N values, or no change at all [140,144].

### 4.4. Temporal differences in isotopic niche and impact of fisheries closure

Many studies have highlighted the positive impact of fisheries closures on local fish populations [145]. Fisheries closures benefit fish and invertebrate populations, and consequently can improve prey availability for top predators. When fishing pressure is relieved, the ecosystem can undergo changes at multiple trophic levels. Removal of top predators can lead to changes in marine community structure such as increases in the population size of lower levels of the trophic web [146]. This has been observed in Alaskan sea otters (*Enhydra lutris*) which have a substantial effect on ecosystem structure by encouraging kelp growth through predation on slow-moving herbivorous invertebrates [147]. This led to enhancement in the overall biodiversity of kelp forests and the promotion of structural complexity in the ecosystem [146]. Conversely, the absence of sea otters has resulted in dense populations of invertebrates such as abalone (*Haliotis* spp.) [148]. This has also been observed in rocky intertidal communities, where bird predators enhanced acorn barnacle abundance (*Balanus glandula*) through the consumption of limpets (*Lottia digitalis*) [149]. Declines in the abundance of 11 species of large sharks have also been associated with concurrent increases in elasmobranch mesoconsumers, which are known prey of sharks [150]. Marine ecosystems may be governed by the strength of trophic links between top predators and lower levels of the food web, relative to other factors, yet there is limited information available describing the effect of fisheries closures on predator–prey interactions. Here, we highlight how the isotopic niche space of Māui dolphins has changed since the introduction of the MMS in 2008.

There is a clear difference in isotopic values between samples collected before and after 2008, and the minimal overlap of ∂^13^C and ∂^15^N values between before 2008 and 2020–2021 indicates niche space has changed substantially between these periods. It is possible the reduction in niche space observed after implementation of the MMS is due to increased prey availability, as the removal of fishing competition is expected to increase the abundance and size of near-shore species [151–153]. This is consistent with other generalist predators where isotopic niche space has reduced inside protected areas. Californian kelp bass (*Paralabrax clathratus*) had smaller isotopic niche spaces inside marine-protected areas (MPAs) compared to those at fished reference sites. This was possibly due to a lower density of high-quality prey outside of MPAs which increased interspecific competition and forced individuals to broaden their diet [154]. Octopuses (*Octopus bimaculatus*) have also shown significantly different isotope values inside and outside of MPAs around Santa Catalina Island, reflecting differing prey contributions between the two areas [155]. Similarly, the isotopic niche space of Magellanic penguins (*Spheniscus magellanicus*) increased in response to decreased abundance of forage fish [156]. However, the decline in Māui dolphin ∂^13^C values over time seen here suggests that dolphins may be consuming more pelagic, offshore sources of prey. Sea-surface temperature (SST) is a major driver of distribution in Māui dolphins [56,66], and it is likely that climate-driven, oceanographic variables such as SST also play a major role in the distribution and abundance of preferred prey, resulting in Māui dolphins foraging further offshore.

Previous work analysing stomach contents from two Māui dolphins showed they feed on red cod, ahuru and sole, similar prey types and sizes to those consumed by Hector's dolphins around the South Island (Te Waipounamu) [65]. In a predictive analysis of the spatial distribution of key prey consumed by Hector's and Māui dolphin, the west coast of the North Island was identified as having considerably lower diversity and biomass of these prey (in addition to three other species consumed by Hector's dolphins) when compared to the remainder of New Zealand [56]. With no commercial fishing and limited amounts of recreational fishing conducted in the core Māui dolphin habitat, there is currently no immediate resource competition with fishing pressures, which is further evidence to suggest that prey availability and abundance are influenced by oceanographic variables [56].

### 4.5. Temporal variation of prey contribution to diet

Here we highlight how Bayesian mixing models were used to demonstrate how Māui dolphin diet has changed over time. We found the prey types consumed by the dolphins were consistent across the years 2010–2011, 2015–2016 and 2020. Throughout this period, diet was primarily comprised of continental shelf-associated prey from benthopelagic origins, indicating this prey type is important for Māui dolphins across all years after 2008. Māui dolphin samples collected before 2008 had much greater ∂^13^C values and slightly increased ∂^15^N values. This suggests dolphin diet prior to 2008 was comprised of more inshore species of a higher trophic level when compared to years following 2008, or the nitrogen and carbon isotopic baseline has shifted due to a change in nutrient input and net productivity, respectively [32]. The overlap of credible intervals between isotopically distinct prey sources confirms Māui dolphins consume a wide variety of prey species throughout the water column and adapt their diet based on available prey. This was not unexpected as this has been observed previously in Hector's dolphins [65].

When assessing dolphin data for suitability to use in mixing models, we observed a proportion of dolphins which fell outside of the mixing space (figure 5). Most of these represented samples collected in the years 2015–2016, which suggested we were missing one or more important prey sources or that dolphins were feeding in an area with elevated nitrogen and carbon baseline values during this period. To reduce the chance of introducing a ‘missing source bias’ in our Set B model [81], and to test how important the absent source(s) were for dolphins in Cluster 2, we artificially extended the mixing space by the inclusion of an undefined, hypothetical source. This approach has been used previously in mass balance models [86,87]. We hypothesize the missing source represents a prey type which has been feeding in an area of elevated nitrogen and carbon baseline. The nitrogen value of the absent source is so high that it is not plausible for this to represent an animal which could be consumed by a Māui dolphin, as they prefer to target small, juvenile prey. Such high nitrogen values have been observed in large, apex predators (e.g. polar bears, sea lions [157,158]). Therefore, we believe this effect to be caused by prey which have which come from an area of elevated nitrogen and carbon baseline. Particularly, the elevated carbon values observed here may suggest an anomalous southern movement of prey from lower latitudes into the habitat frequented by Māui dolphins [34]. Climate-mediated dispersal of several fish species has been reported in New Zealand [159], so it is possible the Cluster 2 individuals have consumed fish from an area of elevated nitrogen and carbon baseline during the 2015–2016 period, which are not usually available otherwise.

### 4.6. Caveats

When conducting mixing model analysis, we have made several assumptions regarding prey species and TEFs. Due to the limited information available on preferred Māui dolphin prey, a wide variety of different species including potential prey were collected and assumed to be representative of the ecosystem. Many of these species adopt a generalist diet, causing their isotopic values to overlap in mixing space. To ensure model source inputs were isotopically distinct, the species had to be grouped based on isotopic values. It was assumed that each resultant source represented a different ecological niche, when in reality the niches are likely to overlap.

The period of prey collection was from 2012 to 2021; in order to assess temporal changes in Māui dolphin diet, we are assuming the isotopic composition of prey remains stable throughout this period. We have no available data to support this assumption but are aware of the importance of tracking such changes. We are now beginning a long-term prey sampling programme for this region to support future trophic studies of Māui dolphins and other coastal species. Given the importance of isotopic temporal variability highlighted in this study, we recommend timeseries sampling and isotopic analysis of baseline organisms to enable isotopic baseline variability to be monitored and accounted for in future studies. TEFs are specific to species and tissue types, and their use in mixing models can have a significant effect on the resultant posterior distribution [80,81]. Due to the absence of an empirically determined TEF for Māui dolphins, we have used a value specific to common bottlenose dolphins (*Tursiops truncatus*).

Given that the Māui dolphin samples were collected between January and March, and the isotopic skin turnover rate of similar cetaceans is estimated to be between two and four months [83], the isotope values presented here are likely to represent prey consumed between September and January during the austral summer/spring. Consequently, our results do not allow us to infer the autumn and winter diets of Māui dolphins. To determine if seasonal variation in diet occurs, a similar study would need to be carried out where samples were collected between June and September.

## 5. Summary and next steps

This work represents an important first step to address knowledge gaps in the foraging ecology of a critically endangered coastal predator, the Māui dolphin. Here we have highlighted that the diet of the Māui dolphin, reflected in the ∂^13^C and ∂^15^N values of skin, has significantly changed over time. Since the implementation of the MMS in 2008, isotopic niche space of Māui dolphins has substantially reduced. The ∂^13^C and ∂^15^N values revealed a decreasing trend with time, except for the years 2015–2016. Isotope values in 2015–2016 were like those observed prior to 2008 and are indicative of a substantial shift in prey distribution and abundance during this time, and/or reflect prey which have come from an area of elevated carbon and nitrogen baseline. This period coincides with the largest El Niño event to occur in the Pacific region in the last 145 years [118], suggesting the distribution and abundance of preferred Māui dolphin prey are affected by climate-driven events.

Long-term genetic monitoring of Māui dolphins has allowed us to interpret isotope values alongside unique DNA profiles. We highlight that the variation observed in 2015–2016 is not due to individual specialization, rather it occurs at the population level. Most of the individuals with elevated nitrogen and carbon values in 2015–2016 were also sampled in 2010–2011, 2020 and/or 2021. In these other years, the isotope values are back within the range of the normal population. Future studies could implement the use of compound-specific stable isotope analysis (CSIA) to disentangle isotopic differences caused by baseline changes, trophic level changes or differences in physiological state [28]. CSIA has been used to prove that variance in nitrogen values cannot be explained by changes in trophic level and therefore likely reflects differences in the isotopic composition at the base of the trophic network [160]. This analysis could provide further insight into the possible causes of elevated nitrogen and carbon values observed in 2015–2016.

Given the increased vulnerability of coastal habitats to climate change and anthropogenic disturbance, the impact that oceanographic variables (e.g. SST and turbidity) have on prey distribution and abundance deserves to be investigated further. Here we have highlighted how climate-driven events such as ENSO may cause substantial shifts in prey distribution, and therefore diet changes in a top marine predator. Climate change poses a significant threat to this critically endangered species; as ENSO is often used as a proxy to predict the future impact of climate change on coastal organisms [45], the relationship between climate change, prey and Māui dolphins warrants further investigation.

## Data Availability

The datasets supporting this article have been uploaded as part of the electronic supplementary material [161].
